# Pneumothorax due to perforated pulmonary hydatic cyst with bronchial fistula

**DOI:** 10.1093/omcr/omaf032

**Published:** 2025-05-28

**Authors:** Amir-Hassan Bordbari, Sepideh Safanavaei, Ramin Rouhani, Ali Sharifpour, Elham Sadat Banimostafavi, Mahdi Fakhar

**Affiliations:** Student Research Committee, School of Medicine, Mazandaran University of Medical Sciences, Sari, Iran; Pulmonary and Critical Care Division, Imam Khomeini Hospital, Mazandaran University of Medical Sciences, Sari, Iran; Department of Thoracic Surgery, Mazandaran University of Medical Sciences, Sari, Iran; Pulmonary and Critical Care Division, Imam Khomeini Hospital, Mazandaran University of Medical Sciences, Sari, Iran; Department of Radiology, Shahid Beheshti Hospital, Qom University of Medical Sciences, Qom, Iran; Iranian National Registry Center for Lophomoniasis and Toxoplasmosis, Toxoplasmosis Research Center, Imam Khomeini Hospital, Mazandaran University of Medical Sciences, Sari, Iran; Department of Medical Microbiology and Immunology, School of Medicine, Qom University of Medical Sciences, Qom, Iran; Mazandaran Registry Center for Hydatid Cyst, Mazandaran University of Medical Sciences, Sari, Iran

**Keywords:** cystic echinococcosis, cell free DNA, Iran, ruptured hydatid cyst

## Abstract

Cystic echinococcosis (CE) is a zoonotic disease caused by *Echinococcus granulosus*, with a high prevalence in Iran, particularly in Mazandaran province. This case report presents a 24-year-old male with pneumothorax resulting from ruptured pulmonary hydatid cysts with bronchial fistulization. The patient presented with persisted dyspnea, productive cough, fever, and mild right thoracoabdominal pleuritic pain, alongside a history of occupational exposure as a sheep keeper. Imaging revealed large cystic masses in lungs and a severe right pneumothorax. Despite initial stabilization with chest tube insertion, surgical intervention was aborted due to intraoperative instability, necessitating pulmonary rehabilitation. The hydatid serology ELISA test was negative, however the cell-free DNA (cfDNA) based PCR biomarker confirmed the diagnosis. This report highlights the diagnostic value of cfDNA in pulmonary CE, particularly when serological tests yield negative results. Early detection and multidisciplinary management are critical for optimal outcomes in pulmonary hydatid cysts complicated by pneumothorax.

## Introduction

Cystic echinococcosis (CE) is a chronic zoonotic disease that is found in many parts of the world, including Iran [1, 2]. This disease is caused by the larval stage of the parasites related to Echinococcus granulosus (*E. granulosus*) [2]. Humans acquire CE by ingesting eggs shed in dog feces via the fecal-oral route [1–3]. These eggs develop into larvae that invade internal organs, such as the liver and lungs—the most commonly affected sites— as well as the spleen, kidneys, brain, eyes, heart, bones, and muscles, where they form cysts [1, 2, 4–6]. However, humans serve as dead-end hosts for the parasite, meaning the disease does not spread further [2].

CE is a significant public health issue worldwide, causing direct and indirect financial burdens from human and livestock infections. The World Health Organization estimates that over 1 million people are affected globally [1, 3]. Iran is a hyperendemic area for this disease, and in a systematic review and meta-analysis, the prevalence of this disease in Iran was estimated at 4.2% [7, 8]. Cases have been reported from all regions of Iran, with the northern province of Mazandaran having one of the highest infection rates [1]. *E. granulosus* is a complex parasite with ten different genotypes identified [2]. In Iran, five genotypes of *E. granulosus* have been found (G1, G2, G3, G6, G7), with G1 being the most common genotype in the World, Iran, and Mazandaran [2, 8, 9].

Although imaging techniques can reveal the characteristic appearances of hydatid cysts, a definitive diagnosis typically relies on serological tests. The enzyme-linked immunosorbent assay (ELISA) is commonly employed for this purpose due to its high sensitivity (approximately 95%), although its sensitivity may drop to around 50% for pulmonary hydatid cysts. It's important to note that the specificity of these tests can vary significantly [10, 11].

This case report highlights a young patient from Mazandaran, northern Iran, presenting with two large pulmonary hydatid cysts. The diagnosis was confirmed using a novel biomarker, circulating cell-free DNA (cfDNA), in clinical specimens, marking one of the few documented cases utilizing this advanced diagnostic approach [12].

## Case presentation

A 24-year-old male arrived at the emergency department (ED) with a two-month history of dyspnea at rest, a productive cough, fever, and myalgia. These symptoms had significantly worsened in the three days leading up to his admission. Notably, the patient had worked as a sheep keeper until three years ago. He also complained of mild right thoracoabdominal pleuritic pain and reported one episode of non-massive hemoptysis two weeks earlier.

Upon arrival, his vital signs were as follows: blood pressure 127/82 mmHg, heart rate 105 bpm, respiratory rate 20 breaths per minute, temperature 36.5° Celsius, and oxygen saturation (SpO2) of 95% on room air. He had a history of smoking and reported no recent contact with ill individuals. Physical examinations revealed decreased respiratory sounds in the right lung, with no other remarkable findings.

**Figure 1:**
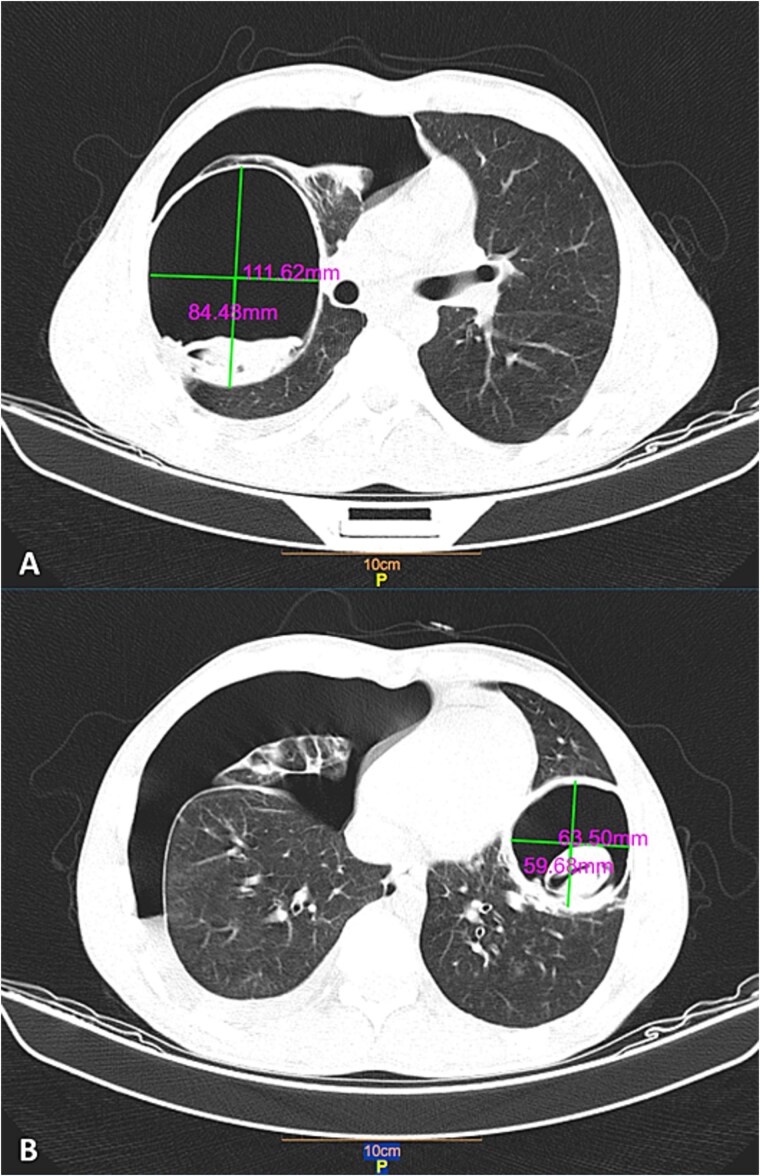
Chest CT scan showing two cystic lesions with smooth, thick-walled structures containing internal densities. The cysts measure 112 × 84 mm (A) and 64 × 60 mm (B) and are accompanied by severe right pneumothorax. The findings collectively indicate the rupture of a hydatid cyst with bronchial fistulization. In images A and B, black arrows indicate the cysts, while white arrows denote the collapsed right lung.

**Figure 2:**
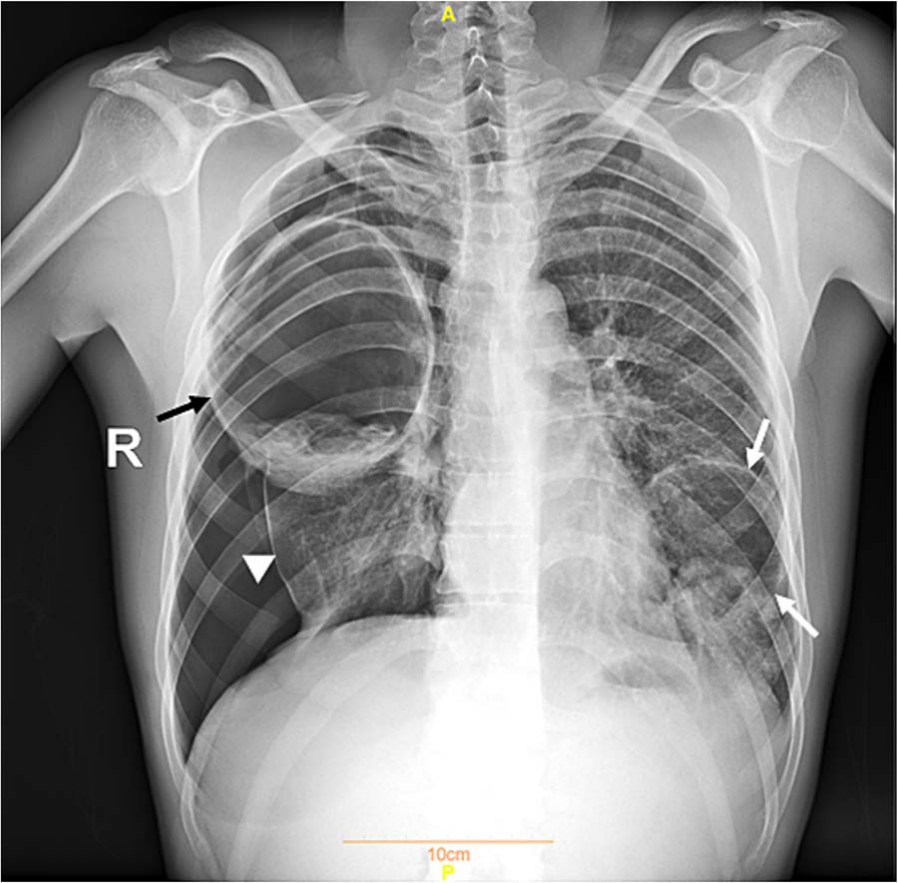
Plain chest radiograph on admission showing a severe right pneumothorax and large, spaceoccupying masses in each lung. The black arrow indicates the cyst in the right lung, while the white arrows denote the cyst in the left lung. The white arrowhead indicates the collapsed right lung.

Chest radiography and computed tomography (CT) scans revealed a severe right pneumothorax and large, space-occupying masses in both lung (Figs 1 and 2). These findings suggested the rupture of a hydatid cyst with bronchial fistulization. To manage the pneumothorax, a chest tube was promptly inserted into the right hemithorax.

Table 1 summarizes the laboratory findings on admission. Serological testing for a hydatid cyst was performed using the commercially available ELISA kit (Pishtaz Teb, *Echinococcus* IgG ELISA, Iran) with a negative outcome. Subsequently polymerase chain reaction (PCR) testing based on circulating cfDNA [12] returned positive for hydatid cyst, with cf DNA detected in serum and urine samples. Consequently, the patient was prescribed albendazole 800 mg daily in three courses of 28 day with 14-day interval for 6 months.

**Table 1:** Baseline laboratory results.

Parameter	Value	Reference value	Unit
Hgb	14.2	11.7–16	mg/dL
PLT	265	130–400	10^3^/mm^3^
WBC	13.0	4.5–11	10^3^/mm^3^
Poly	60.1	37–72	%
Lymph	15.5	20–50	%
MIX	24.4		%
CRP	80.0	Up to 6	mg/dL
ESR	18	0–15	mm/hour

Hgb: hemoglobin, PLT: platelets, WBC: white blood cells, Poly: polymorphonuclear leukocytes, Lymph: lymphocytes, MIX: Mixed Cell Population, combined percentage of monocytes, eosinophils, and basophils, CRP: c-reactive protein, ESR: erythrocyte sedimentation rate

**Figure 3:**
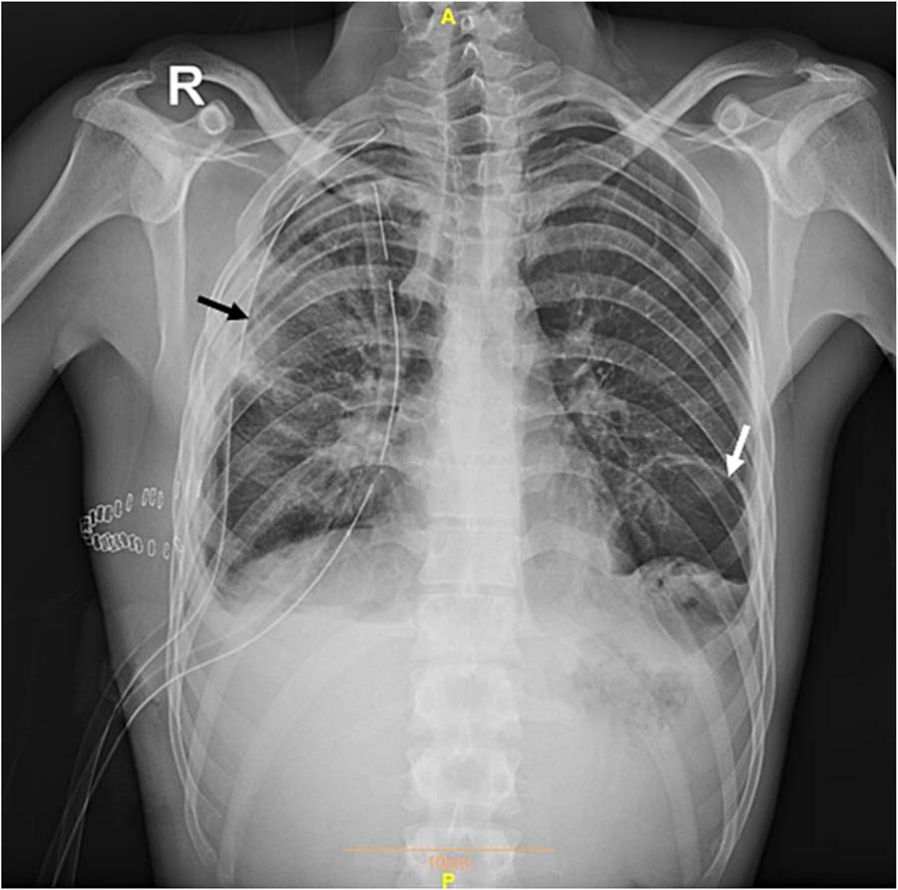
Postoperative plain chest radiograph showing successful removal of the right hydatid cyst. The black arrow indicates the residual opacity at the site of the previous lesion in the right lung, while the white arrow denotes the cyst in the left lung. Two chest tubes are visible in the right hemithorax.

Following four weeks of albendazole therapy, the patient was scheduled for hydatid cyst removal surgery, as per standard treatment protocols. However, shortly after intubation and initiation of one-lung ventilation, the patient's vital signs became unstable, and oxygen saturation dropped. As a result, the surgery was promptly canceled, and the patient was referred for pulmonary rehabilitation. Following four weeks of rehabilitation, the cysts were successfully removed in two separate surgeries (Fig. 3 shows the patient after the first cyst removal). The patient remained stable postoperatively, with no complications during follow-up visits.

## Discussion

In this case report, we present a young patient with a history of cough and worsening shortness of breath, who was found to have hydatid cysts in both lungs, along with a spontaneous pneumothorax. The patient reported occupational exposure in previous years. Following an unsuccessful attempt at surgery, the cysts were successfully removed, and the patient showed good outcomes during follow-up. Notably, in light of the negative serological results, circulating cfDNA-based PCR was utilized to confirm the diagnosis of hydatid cysts before surgery. This case highlights that a negative serological test does not rule out the possibility of CE, especially in pulmonary cases.

Hydatid cyst disease is closely associated with occupational exposure and is more frequently observed among shepherds, livestock keepers, butchers, and veterinarians who have direct contact with animals [2]. It is worth mentioning that our patient had a history of being a sheep keeper three years prior to admission to the hospital. Mazandaran province in northern Iran, characterized by a moist climate and moderate temperatures, has reported one of the highest infection rates [1, 5]. Recently, A registry-based study conducted at our center highlights a rising trend in hydatid cyst diagnoses over the past two decades [1]. Given the proximity of Mazandaran to Tehran, the capital of the country and a hub of scientific centers and academic hospitals, it is estimated that these figures are just the tip of the iceberg [1].

Hydatid cysts are often asymptomatic and can grow slowly over several years without being diagnosed [4, 6]. Pulmonary cysts tend to grow more rapidly compared to hepatic cysts [2]. Hydatidosis presents with diverse symptoms, depending on the location, size, and pressure effect of the cyst on adjacent organs [4]. Symptoms usually manifest when the cyst reaches a sufficient size or becomes complicated [2]. Cysts larger than 5 centimeters may exert pressure on the bronchus [2, 6]. Pulmonary hydatid cysts are always at risk of rupture, which can lead to allergic manifestation like urticaria, asthma, or even anaphylaxis [2, 4, 6, 13]. Factors like coughing, trauma, increased cyst size (7–10 cm), or elevated intrathoracic pressure can trigger rupture [3, 13]. Rupture may extend into the pleura or bronchus. When ruptured into the pleura, it can result in pleural effusion, infection, empyema, or pneumothorax. Pneumothorax can also arise from a ruptured cyst with a bronchial fistula. Acute symptoms such as cough, hemoptysis, fever, chest pain, and others may indicate cyst rupture [2, 6, 13].

When evaluating suspected hydatid cysts, it is crucial to rule out other differential diagnoses, including benign cysts, mycoses, abscesses, cavitary tuberculosis, and benign or malignant neoplasms [4]. Imaging techniques, including X-ray, CT, and magnetic resonance imaging (MRI), are among the most useful tools for detecting pulmonary CE [3, 4]. Laboratory tests further support diagnosis [3]. A promising biomarker for human CE is circulating cfDNA, which can be detected in serum, urine, and bronchoalveolar lavage (BAL) samples. This biomarker is useful for both diagnosis and treatment monitoring [14, 15].

Effective control of CE in endemic areas requires comprehensive preventive strategies. These include maintaining environmental hygiene and conducting education and awareness campaigns. Key preventive measures involve the safe disposal of animal offal, restricting domestic dogs' access to slaughterhouse waste, avoiding direct contact with dog feces, enhancing meat inspection regulations, and providing veterinary services such as vaccination and treatment for infected animals [1, 8].

## Conclusion

This case highlights the critical need for early diagnosis and a multidisciplinary approach in managing pulmonary hydatid cysts complicated by pneumothorax. It should be noted that a negative serological test does not rule out the diagnosis of CE, particularly in cases of pulmonary CE. Therefore, it is highly recommended to combine multiple diagnostic modalities, including cfDNA-based PCR on various clinical specimens, to enhance diagnostic accuracy. Preventive strategies, including education, environmental hygiene, and veterinary measures, are crucial in endemic areas to reduce the incidence and complications of cystic echinococcosis.

## Consent

Informed consents for publication of this case series were taken from the patients.

## Guarantor

Mahdi Fakhar is the guarantor of this article.
